# Application of the Solute–Solvent Intermolecular Interactions as Indicator of Caffeine Solubility in Aqueous Binary Aprotic and Proton Acceptor Solvents: Measurements and Quantum Chemistry Computations

**DOI:** 10.3390/ma15072472

**Published:** 2022-03-27

**Authors:** Tomasz Jeliński, Maciej Kubsik, Piotr Cysewski

**Affiliations:** Department of Physical Chemistry, Pharmacy Faculty, Collegium Medicum of Bydgoszcz, Nicolaus Copernicus University in Toruń, Kurpińskiego 5, 85-950 Bydgoszcz, Poland; chemfiz@cm.umk.pl

**Keywords:** caffeine, solubility, green solvents, aprotic proton acceptor solvents, 4-formylmorpholine, affinity, hetero-molecular complexes, COSMO-RS

## Abstract

The solubility of caffeine in aqueous binary mixtures was measured in five aprotic proton acceptor solvents (APAS) including dimethyl sulfoxide, dimethylformamide, 1,4-dioxane, acetonitrile, and acetone. The whole range of concentrations was studied in four temperatures between 25 °C and 40 °C. All systems exhibit a strong cosolvency effect resulting in non-monotonous solubility trends with changes of the mixture composition and showing the highest solubility at unimolar proportions of organic solvent and water. The observed solubility trends were interpreted based on the values of caffeine affinities toward homo- and hetero-molecular pairs formation, determined on an advanced quantum chemistry level including electron correlation and correction for vibrational zero-point energy. It was found that caffeine can act as a donor in pairs formation with all considered aprotic solvents using the hydrogen atom attached to the carbon in the imidazole ring. The computed values of Gibbs free energies of intermolecular pairs formation were further utilized for exploring the possibility of using them as potential solubility prognostics. A semi-quantitative relationship (R^2^ = 0.78) between caffeine affinities and the measured solubility values was found, which was used for screening for new greener solvents. Based on the values of the environmental index (EI), four morpholine analogs were considered and corresponding caffeine affinities were computed. It was found that the same solute–solvent structural motif stabilizes hetero-molecular pairs suggesting their potential applicability as greener replacers of traditional aprotic proton acceptor solvents. This hypothesis was confirmed by additional caffeine solubility measurements in 4-formylmorpholine. This solvent happened to be even more efficient compared to DMSO and the obtained solubility profile follows the cosolvency pattern observed for other aprotic proton acceptor solvents.

## 1. Introduction

**Figure 1:**
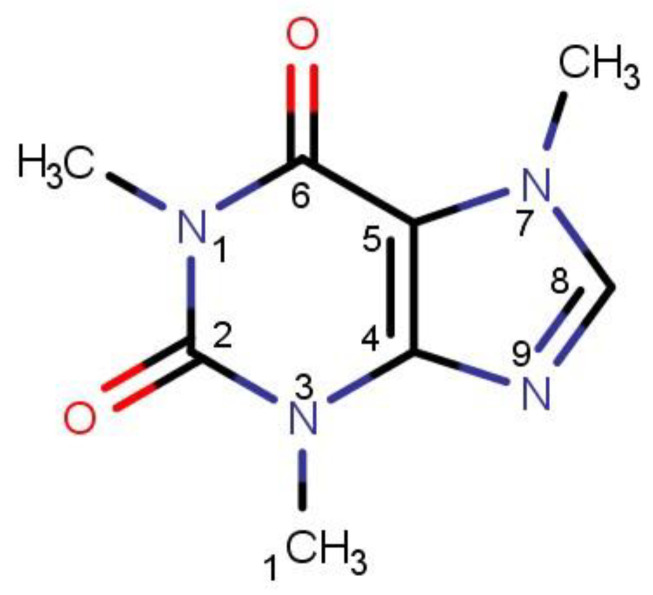
The structural formula of caffeine with atom labeling in the pyridine and imidazole rings.

Methylxanthines, belonging to a broad range of alkaloids, are an interesting group of compounds experiencing significant pharmacological activity. From the perspective of the pharmaceutical industry, there are three most important compounds in this category, namely theophylline (1,3-dimethylxanthine) [1], theobromine (3,7-dimethylxanthine) [2] and caffeine (1,3,7-trimethylxanthine). Methylxanthines are abundant in nature residing in tea and other plant leaves, coffee and cocoa beans, as well as cola seeds [3]. From a chemical perspective, methylxanthines are alkaloids based on purine, which is a fused heterocyclic system containing both pyrimidine and imidazole rings [4]. Methylxanthines are generally fairly poorly soluble in water and ethanol [5] and have a low water–octanol partition coefficient [6]. While theophylline and theobromine are amphiprotic compounds, caffeine does not possess acidic properties [7] due to the methylation of all heteroatoms. Methylxanthines are readily absorbed in the gastrointestinal tract and can penetrate to the central nervous system where their psychostimulating properties are visible. The activities of methylxanthines include stimulation of the central nervous system, increase of blood pressure, diuresis of kidneys, relaxation of smooth muscles, strengthening of the concentration of skeletal muscles or gastric acid secretion [8,9]. These activities are the result of the ability of methylxanthines to act as phosphodiesterase inhibitors [10] and nonselective adenosine receptor antagonists [11]. Caffeine (structural formula presented in Figure 1), one of the three most pharmacologically important methylxanthines as mentioned earlier, is found in many plants traditionally associated with the presence of methylxanthines [12,13]. It has been consumed by humans for thousands of years with the earliest accounts dating back to ancient China [14,15], from which the custom of using this compound in the form of brewed tea and coffee would spread around the world, although caffeine was isolated for the first time in 1819 and synthesized in 1865 [16]. Nowadays it is estimated that about 80% of the world’s adult population consumes 200–250 mg of caffeine every day [17,18]. The absorption of caffeine from the small intestine after oral intake is rapid and complete [19] and its elimination from the organism occurs mainly through excretion in urine with paraxanthine being the main metabolite [20]. The mode of action of caffeine is related to binding to adenosine receptors [21] and inducing psychomotor stimulant properties in the brain [19]. The beneficial effects of caffeine comprise improved alertness, mood and performance, reduced fatigue and alleviated effects of sleep deprivation [22,23,24], although its excessive consumption can lead to several side effects [21,25,26].

The solubility of different active pharmaceutical ingredients (APIs) is a crucial parameter utilized in the pharmaceutical industry and their inadequate solubility is a well-recognized limitation [27] in the development of new drugs. For example, according to BCS (Biopharmaceutics Classification System) criteria, as many as 40% of drugs can be regarded as practically insoluble in water [28] and this number is even higher for newly developed drugs [29]. There are many strategies used to overcome this difficulty including amorphization [30,31], formation of monocrystals [32,33], micronization [34] and solid dispersion formation [35], complexation with cyclodextrins [36,37], salts [38,39] and cocrystals formation [40,41]. Using cosolvation techniques is another way of increasing solubility. The cosolvation effect takes place when a particular amount of a cosolvent is added to the primary solvent and causes a solubility increase [42,43]. Different organic compounds can be used for this task, including DMSO, ethylene glycol, propylene glycol, glycerin, ethanol and others [44]. Studies show that the usage of cosolvents may lead to a solubility increase of even several hundred times [45]. The mechanism of solubility improvement due to cosolvation is still extensively studied. Generally speaking, cosolvency increases the solubility of hydrophobic/lipophilic drugs by disturbing the intermolecular network of hydrogen bonds present in aqueous systems. This in turn lowers the polarity of the system and creates an environment with physicochemical properties closer to those of the drug, according to the general rule that “similar dissolves in a similar” [46]. Very important in the context of the practical application of cosolvation techniques are the considerations regarding the design of cosolvent systems and their influence on drug solubility [44,47,48,49] often combined with other strategies addressing low drug solubilization. These can include the pH modification of the solution [50], the use of surfactants [51,52] or complexation with cyclodextrins [53,54].

Caffeine solubility was extensively studied experimentally in neat organic solvents [55,56,57], binary solvent mixtures [58,59,60,61,62,63,64,65,66,67] and carbon dioxide [68,69]. It is interesting to note that in several binary solvents a cosolvency effect was observed. For example, mixtures of methanol + carbon tetrachloride [60] and ethyl acetate + ethanol [61] offer a significant cosolvency effect. Furthermore, aqueous mixtures of methanol [62], ethanol [61], dimethyl formamide [60] or 1,4-dioxane [63] exhibit similar behavior. To the contrary, such a phenomenon is not observed for systems comprising carbitol + ethanol [64] and N-methyl-2-pyrrolidone in mixtures with ethanol [58], n-propanol [65], isopropanol [66], ethylene glycol [59] or propylene glycol [67].

Apart from traditional organic solvents, ionic liquids and deep eutectic solvents are gaining increasing attention and utilization in the context of dissolution and extraction of active pharmaceutical ingredients [70,71]. This is due to their beneficial properties including excellent solubilizing potential, environmental friendliness and relatively low cost of preparation. Additionally, the behavior of caffeine in these designed solvents was studied [72,73,74,75,76]. Particularly interesting are the results of caffeine extraction from spent coffee grounds using cholinium-based ionic liquids [72]. Our research team has previously published the results of theophylline and theobromine solubilized in natural deep eutectic solvents utilizing choline chloride [1,2] and similar results regarding caffeine will follow.

The aim of this paper is fourfold. First of all, the total pool of caffeine experimental solubility data is extended by including aprotic proton acceptor solvents (APAS), which were used in binary mixtures with water. Secondly, the observed solubility trends are interpreted in terms of intermolecular interactions quantified using an advanced level of quantum chemistry computations. Thirdly, the gathered information is used for screening with the purpose of finding more environmentally friendly and effective solvents. Finally, the hypothesis formulated at the screening stage is validated by additional solubility measurements aimed at increasing the environmental safety of caffeine dissolution.

## 2. Materials and Methods

### 2.1. Materials

The main compound used in this study was caffeine (CAS: 58-08-2, MW = 194.19 g/mol) purchased from Sigma Aldrich, Saint Louis, MO, USA, with a purity of ≥99%. All of the utilized organic solvents were supplied by Avantor Performance Materials, Gliwice, Poland. These were the following: methanol (CAS: 67-56-1), dimethyl sulfoxide-DMSO (CAS: 67-68-5), dimethylformamide-DMF (CAS: 68-12-2), 1,4-dioxane (CAS: 123-91-1), acetonitrile (CAS: 75-05-8), acetone (CAS 67-64-1) and 4-formylmorpholine (CAS: 4394-85-8). All solvents had a purity of ≥99% and were used without any initial procedures.

### 2.2. Preparation of the Calibration Curve

In order to obtain a calibration curve for caffeine, its stock solution was prepared with a concentration equal to 3.23 mg/mL in a 100 mL volumetric flask using methanol as a solvent. Then, successive dilutions were made by transferring specific volumes of the stock solution into 10 mL volumetric flasks and diluting them with methanol, thus obtaining a series of caffeine solutions with decreasing concentration. Spectrophotometric measurements of such solutions were conducted and the values of absorbance at 272 nm wavelength were plotted against the concentration values. Three separate series of measurements were performed and the final curve resulted from averaging. The statistical analysis of the obtained curve included the determination coefficient R^2^, limit of detection (LOD) and limit of quantification (LOQ). Details regarding the calibration curve can be found in Supplementary Materials in Table S1a,b.

### 2.3. Preparation of Samples in Aqueous Binary Mixtures of Organic Solvents

For preparing the solvent mixtures utilized in the study, preset amounts of the organic solvent and water were mixed together in 10 mL volumetric flasks with different mole ratios. Next, excess amounts of caffeine were placed in test tubes which were then filled with the solvent mixture prepared earlier or with a neat solvent. This way saturated solutions of caffeine were obtained. The prepared samples were incubated for 24 h at different temperatures using an Orbital Shaker Incubator ES-20/60 supplied by Biosan, Riga, Latvia. The temperature adjustment accuracy was 0.1 °C and its variance during the daily cycle was ±0.5 °C. The mixing of the samples at 60 rev/min was provided by the incubator. After incubation, the samples were filtered with the help of a syringe equipped with a PTFE filter with 22 µm pore size. All of the test tubes, syringes and filters were preheated at the appropriate temperature to avoid precipitation of dissolved caffeine. Next, fixed amounts of the filtrate were transferred to test tubes filled with a set amount of methanol and the diluted samples were measured spectrophotometrically. In order to establish the mole fractions of caffeine in solutions, the density of the samples was determined.

### 2.4. Solubility Measurements

The caffeine concentration in the samples was measured spectrophotometrically with the use of an A360 spectrophotometer from AOE Instruments, Shanghai, China. The procedure involved obtaining spectra in the 190–700 nm wavelength range with a 1 nm resolution and utilizing the absorbance values at 272 nm. Since methanol was used for diluting the samples it was also used as a reference during spectrophotometric measurements. Furthermore, in order for the absorbance to remain in the linearity limit of the calibration curve, the samples were diluted accordingly using methanol. Three samples were measured for each system and their concentrations were averaged and expressed as mole fractions.

### 2.5. FTIR–ATR Measurements

Solid sediments of caffeine were analyzed by FTIR-ATR measurements after collecting residues from the test tubes after solubility experiments described above. The samples were dried and measured using an FTIR Spectrum Two spectrophotometer from Perkin Elmer, Waltham, MA, USA, equipped with a diamond attenuated total reflection (ATR) device. The spectra were recorded in the 450–4000 cm^−1^ wavenumber range.

### 2.6. Differential Scanning Calorimetry Measurements

The characterization of caffeine behavior in the presence of water, organic solvents and their mixtures was conducted using the differential scanning calorimetry (DSC) technique. Firstly, 0.4 g of caffeine samples were ground with appropriate amounts of different solvents using a MM 200 mill from Retsch, Haan, Germany. The frequency was set to 25 Hz and the grinding lasted for 30 min. Jars (5 mL) from stainless tell were used for grinding the samples with two stainless steel balls. For calorimetric measurements, the DSC 6000 from PerkinElmer, Waltham, MA, USA, was used with a heating rate of 10 K/min and 20 mL/min nitrogen flow for providing an inert atmosphere. Standard aluminum pans were used for the measurements and the calorimeter was initially calibrated with indium and zinc standards.

### 2.7. Affinity Quantum Chemistry Computations

Although solubility can be sometimes explained in terms of solvation energy [77], we can offer a much better relationship between computed values of Gibbs free energies and experimental solubility, even though it seems that such trend is of local character and holds for a given compound or a class of compounds in a limited range of solvents. Therefore, in this work, we have interpreted the solubility of caffeine in terms of solute–solvent intermolecular interactions.

Intermolecular interactions of caffeine were modeled using an advanced quantum chemistry level including electron correlation corrections and zero-point vibrational energy ZPE. The computational protocol follows an already presented methodology [2,78,79,80,81] and here only a brief summary is provided. It is assumed that the solute–solvent affinity between constituents of considered binary solvents can be expressed, as the first approximation, in terms of corresponding reactions of pair formation, namely X + Y = XY, where X and Y stand either for solute or solvent molecules. In the case of X = Y dimers are formed of AA, BB1 or BB2 type, where A stands for caffeine, B1 for organic solvent and B2 represents a water molecule. The other three hetero-molecular complexes, namely AB1, AB2 and BB12, complete the variations of all possible binary contacts. The practical calculations of the thermodynamic properties require a reasonable representation of the molecular structure both in the gas and in the condensed phases. Since many molecules can exist in a variety of conformers, which can affect the structure of bimolecular contacts, extensive conformational screening of biomolecular systems was performed. Many potential bimolecular clusters were considered by automatic generation of structures with the highest probability of interactions between the surface segments of molecules, which is offered by the COSMOtherm program [82] via invoking the command “CONTACT = {1 2} ssc_probability ssc_weak ssc_ang = 15.0”. This typically leads to a quite large number of potential structures, which further undergo geometry optimization and clustering. The optimization of structures was carried out using RI-DFT BP86 (B88-VWN-P86) with def-TZVP basis set, which was followed by single-point energy computations using def2-TZVPD basis set with the fine grid tetrahedron cavity and inclusion of parameter sets with hydrogen bond interaction and van der Waals dispersion term based on the “D3” method of Grimme et al. [83]. All quantum chemistry calculations were performed using TURBOMOLE version 7.5.1 [84] interfaced with BIOVIA TmoleX version 21.0.1. [85]. The values of Gibbs free energies were determined using COSMOtherm version 21.0.0 [82] with BP_TZVPD_FINE_21.ctd parametrization.

**Scheme 1:**
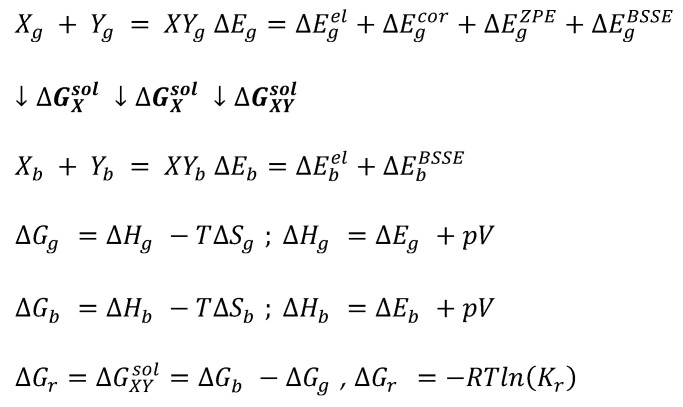
The thermodynamic cycle used for computations of the values of Gibbs free energy of solute–solvent affinities represented by chemical reaction. Subscripts *g* and *b* denote the gas and bulk phases, respectively.

Computations of the equilibrium constant are realized in COSMOtherm via a typical thermodynamic cycle scheme. It involves geometry optimization both in the gas and bulk phases followed by thermal free energy contribution determined via the COSMO-RS approach, as summarized in Scheme 1. The thermodynamic characteristics of the reaction in the gas phase is the first step, which critically determines the accuracy of predicted values of equilibrium constant. It is essential to emphasize that currently COSMOtherm parameterizations are restricted to density functional theory (DFT) models, which determine the accuracy of the reaction energy. In particular, the BP86 functional is used in the majority of physical properties computations. Due to its inherent limitations, it is necessary to go beyond DFT using high-level quantum chemistry computations for enhancing the accuracies of energy determination. Hence, three types of corrections to the values of electronic energy are added for accurate computations of the total gas phase energy, namely (1) accounting for the electron correlation energy,$\;\Delta E_{g}^{cor}$, improperly computed using merely the DFT level, (2) correction for the zero Kelvin vibrational energy,$\;\Delta E_{g}^{ZPE}$, and (3) basis superposition error. Since the gas phase properties can be significantly affected by the accuracy of computations [86], the electron correlation computed on RI-MP2 level of theory was included using an extended Ahlrichs def2-QZVPP basis set [87] utilizing the geometry determined in the previous step, namely RI-DFT BP86/def-TZVP. Besides, the values of the zero-point vibrational energy (ZPE) were computed at RI-DFT BP86 (B88-VWN-P86)/def2-TZVPD level. The BSSE can be estimated using a DFT-C approach, which formulation includes atom–atom many-body corrections and is a parameterized geometry-based method [88]. These computations are to be performed for every conformer for all reagents and averaged with weights corresponding to population percentage estimated using Boltzmann probability. The final values of the reaction Gibbs free energy, Δ*G_r_*, account for all thermal free energy of the gas phase contribution and the Gibbs free energy of solvation quantifying the contribution of transfer from gas to liquid.

Finally, it is worth adding that in COSMOtherm it is possible to split the contribution to the equilibrium constant into the concentration-dependent contribution, *K_x_*, and activity constant, *K_γ_*, accounting for nonideality. The product of these constants leads to activity and therefore concentration-independent constant, *K_a_*. Hence, for computation of this quantity, the knowledge of the actual concentration of reagents is not necessary. Practically it can be achieved by invoking the command “K_activity” in COSMOtherm computations.

## 3. Results and Discussion

### 3.1. Solid State Characteristics

Caffeine exists in two stable polymorphic forms (termed I and II) and a hydrate, which have been a topic of many studies [89,90,91,92,93]. Form II is stable at temperatures below 151 °C, while form I is stable at higher temperatures up to the melting point of 238 °C. The hydrate on the other hand is subjected to water loss at 145 °C. Additionally, a third polymorphic form was obtained through sublimation [94]. In order to find which forms are present in the solid state obtained after the solubility measurements, both DSC and FTIR techniques were used, as described in the methodology section. Although extensive studies have been performed, here only a selection of results was presented in order to provide a general perspective on the caffeine polymorphic forms present in the samples.

The FTIR measurements have shown that caffeine exists in an anhydrous form in neat solvents, while the addition of water results in obtaining a hydrate. This is exemplified in the case of caffeine dissolved in water–DMSO mixtures, as shown in Figure S1 in Supplementary Materials. The presented IR spectra include pure caffeine, as well as the precipitates obtained after solubility experiments involving water, neat solvent and their mixtures. Pure caffeine and the solid residue obtained after dissolution in neat DMSO (x = 1.0) are basically identical. The characteristic absorption bands of this form involve C-H (alkyl) at around 2900 cm^−1^, C=O at around 1650 and 1710 cm^−1^, C=C at around 1550 cm^−1^ and C-N at around 1250 cm^−1^. However, when caffeine was dissolved in water and in mixtures containing water (x = 0.2 to x = 0.8) an additional new wide band appears with a maximum at around 3400 cm^−1^, which can be attributed to the O-H stretching in the hydrate [95]. The above pattern was repeatedly observed for all studied solvents.

When taking into account the DSC measurements of the studied samples, a similar picture emerges as it was in the case of FTIR studies. Again, for practical purposes, the results obtained for DMSO–water mixtures are presented in Figure S2 in Supplementary Materials. The melting point of pure caffeine, as well as the precipitates, was found in all cases at around 238 °C, with a mean value of 238.32 °C (standard deviation, SD = 0.39 °C) for the whole dataset, which is in good accordance with literature data. However, there are differences in the position of the first endothermic peak between the samples. In the case of pure caffeine and the sample obtained after dissolution in neat solvents a peak with a mean value of 157.87 °C (SD = 0.38 °C) can be observed, which can be attributed to the transition between forms II and I, although it is shifted towards higher temperatures. For the samples obtained after caffeine solubilization in mixtures containing water, a peak with a maximum of 151.66 °C (SD = 0.55 °C) was found, which again is slightly shifted compared with literature but can be assigned to the water loss of the caffeine hydrate. All the above observations indicate that the presence of water in the solvent mixtures results in the presence of the caffeine hydrate, while the lack of water is responsible for the presence of its polymorphic form II.

### 3.2. Experimental Solubility of Caffeine

Before the actual solubility experiments, in order to confirm the reliability and adequacy of the used methodology, some of the results were compared with literature data. When analyzing these results it became evident that there are some discrepancies in the data provided by different authors. However, the results obtained in this study are quite close to the ones obtained by Shalmashi et al. [56] and Dabir et al. [57] with slightly larger differences when compared with Zhong et al. [55]. Detailed results of this comparison can be found in Table S2 in Supplementary Materials, where the mole fractions of caffeine in various solvents at different temperatures were presented together with relative differences between datasets. This analysis, supported by the fact that the results obtained for the two other methylxanthines [1,2] match literature data, suggest the adequacy of the method used in the study.

**Figure 2:**
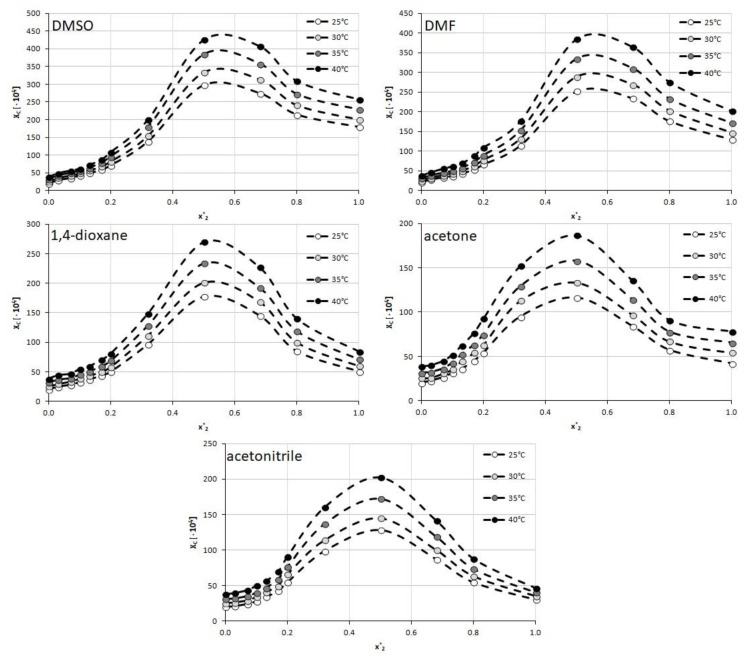
Solubility of caffeine, expressed as its mole fractions, in binary solvents comprising water and one of five studied aprotic proton acceptor solvents in varying compositions. On the abscissa, x_2_^*^ represents the mole fractions of organic solvent in solute-free binary solutions.

The five mentioned organic solvents were used to create binary solvents with water in different mole proportions. For each solvent–water system, twelve different compositions were studied including both neat solvent and water. The temperature range was set from 25 °C to 40 °C with 5 °C intervals. For all of the studied solvents, the increasing temperature resulted in an increase of caffeine solubility and the general trend of solubility profiles remains the same in all cases. The obtained results are collected in Figure 2 and the corresponding detailed values of caffeine mole fractions can be found in Supplementary Materials, Tables S3–S7.

When using DMSO as a cosolvent with water a gradual increase in solubility is observed up to the point corresponding to unimolar proportions of water and DMSO (x_2_^*^ = 0.5) with a less pronounced decrease afterward. The highest value of caffeine solubility at this point was equal to x_C_ = (425.89 ± 0.92)∙10^−4^ at 40 °C which is 65% more than for the neat organic solvent.

The trend of solubility changes observed for DMF is rather similar to that of the former solvent. Again, after an increase in caffeine solubility, an inflection point can be observed at x_2_^*^ = 0.5 with the highest mole fraction equal x_C_ = (383.96 ± 2.16)∙10^−4^ at 40 °C, which stands for a 90% increase in solubility compared to neat DMF. In both of the above solvents, the solubility decline after reaching the point of the optimal composition is less pronounced than the initial increase of solubility.

When taking into account 1,4-dioxane as a cosolvent, somewhat similar behavior of the caffeine solubility profile is observed. Again the unimolar proportions of water and the organic solvent are responsible for the largest solubility of caffeine. In this case the mole fraction obtained at 40 °C for x_2_^*^ = 0.5 was x_C_ = (269.77 ± 2.59)∙10^−4^. Contrary to DMSO and DMF however, the later decrease of caffeine solubility is much more evident as the highest solubility value is more than 3 times larger than the solubility obtained for pure 1,4-dioxane.

Furthermore, in the case of acetone, the highest solubility of caffeine was observed when the proportions of water and the organic solvent were equal to each other. Here, the highest solubility found at 40 °C equaled x_C_ = (186.51 ± 0.95)∙10^−4^, which stood for a more than 2 times increase in solubility when compared to neat acetone. Interestingly and contrary to the three former solvents, the composition of x_2_^*^ = 0.32 was also responsible for quite high values of caffeine solubility, larger than the ones obtained at x_2_^*^ = 0.68. Therefore, the interval of particularly elevated solubility values of caffeine was also wider than in the former cases.

While the overall trend of caffeine solubility changes for acetonitrile is rather similar to the one observed for acetone, there is one interesting feature of the obtained results. The highest solubility found for x_2_ = 0.5 at 40 °C was equal to x_C_ = (202.40 ± 1.88)∙10^−4^ which is higher than for acetone. This means that although pure acetonitrile experiences lower caffeine solubility than pure acetone, the binary mixture of acetonitrile and water is more efficient than the corresponding binary solvent involving acetone. Furthermore, the solubility obtained at the point of the optimal composition is more than 4 times higher than the one obtained for the neat solvent, which is the highest among studied solvents.

### 3.3. Caffeine Interactions in Studied Solvents

Observed solubility trends can be interpreted in terms of intermolecular interactions. For this purpose, the affinities of caffeine toward hetero-molecular pairs formation were calculated using quantum chemistry computations on an advanced level, as described in the methodology section. Selected structural and energetic properties are collected in Table 1.

**Figure 3:**
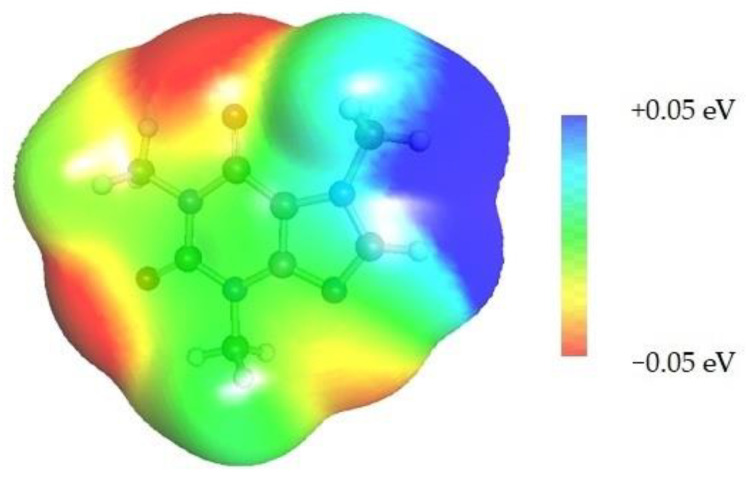
Electrostatic molecule potential color-coded on density isosurface of caffeine representing the range from −0.05 eV to +0.05 eV. Colors indicate electronegative (red) and electropositive (blue) regions.

Quite surprising conclusions can be drawn from the results provided in Table 1, namely, caffeine can act as a donor in pairs formation with all considered APAS. The hydrogen atom bound to the carbon atom within the five-membered ring (C_8_) has quite an acidic character and can be directly involved in non-covalent interactions with solvent molecules. Hence, negative centers located on oxygen atoms of solvent molecules can complete the pair formation with proton-accepting sites. On the contrary, with proton donating molecules, for example, water, caffeine plays the role of an acceptor in pair formation via the basic center of the nitrogen atom (N_9_) located on the imidazole ring. The same feature might be observed for alcohols and other proton donating molecules. In the case of caffeine contacts with aprotic solvents, molecules of the formed pairs can be considered as examples of weak hydrogen bonds. This can be interfered with by geometric features of the considered pairs. It is evident that hydrogen bonds are much longer in all cases of apolar solvents if compared with the caffeine–water hydrogen-bonded pair. Despite this fact, the stabilization of pairs of DMF and DMSO with caffeine is as high as in the water complex. This is in interesting correspondence with the high solubility of caffeine in these solvents. The interactions of caffeine with the other three apolar solvents are weaker, with the smallest stabilization of pairs formed with acetonitrile, and this order also corresponds to the observed solubility. It is also worth highlighting that the involvement of the C_8_ atom of caffeine might be anticipated after inspection of the electrostatic potential distribution. This is documented in Figure 3 by plotting the isosurface in the range of <−0.05 eV, +0.05 eV>. It is clearly visible that there is a very large blue region in the vicinity of the hydrogen atom involved in pairs formation with aprotic molecules, indicating an electropositive character of this fragment of caffeine. Hence, this observation might be used in anticipating interactions with apolar solvents with negative centers.

### 3.4. Solubility Interpretation in Terms of Caffeine Intermolecular Interactions

**Figure 4:**
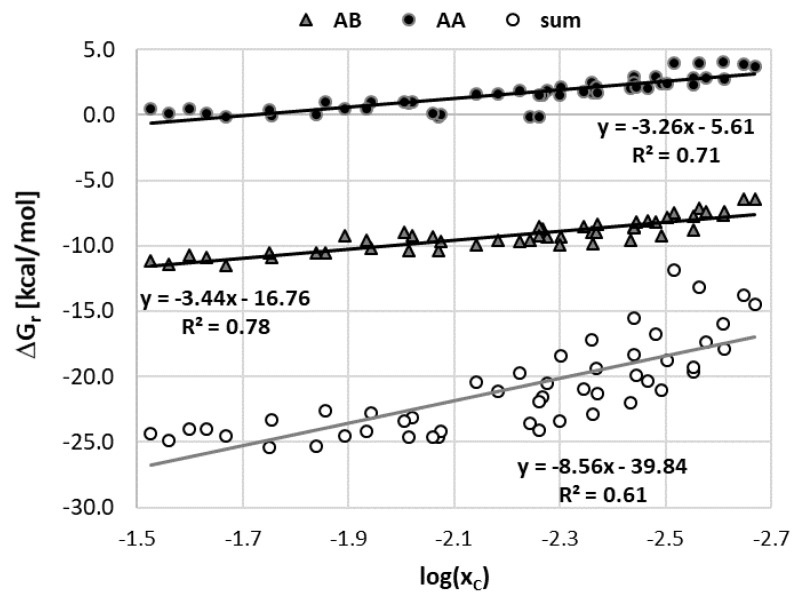
The correlations between the experimental caffeine solubility in binary water-organic mixtures at 25 °C and the solute–solute (AA) affinities, solute–solvent (AB = AB1 + AB2) affinities and total affinities including solvent–solvent interactions (AA + AB + BB), where A stands for caffeine, B1 denotes the organic solvent, B2 denotes water and BB = BB1 + BB2 + BB12.

Previously mentioned qualitative agreement between the computed solute–solvent affinities and measured solubility of caffeine is the starting point for further exploration of the usefulness of the computed values of caffeine affinities as a potential prognostics of its solubility. For this purpose, plots were prepared relating the experimental solubility with computed values of Gibbs free energies of self- and hetero-association of solute–solute (AA), solute–solvent (AB) and solvent–solvent (BB) interactions. The former includes only caffeine dimerization. The second type comprises two kinds of contributions coming from interactions with water and the organic solvent. The latter type involves all homo- and hetero-pairs formed between solvent molecules. The obtained results are provided in Figure 4. In particular, AA denotes caffeine dimerization, AB1 caffeine-organic solvent pairs formation, AB2 caffeine-water pairs formation, BB1 organics self-association, BB2 water dimerization and BB12 hetero-association of solvent molecules.

**Figure 5:**
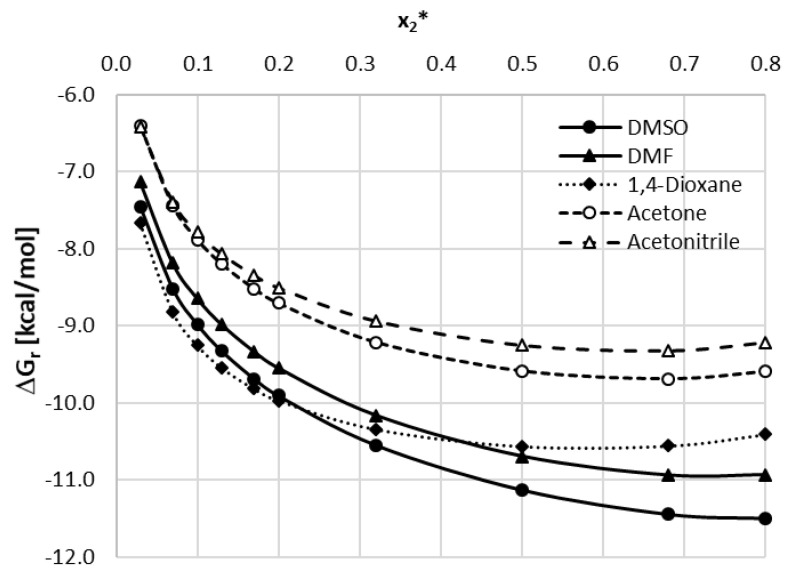
The correlations between the caffeine affinity computed at 25 °C and the composition of the binary solvent for the studied APAS.

The results provided in Figure 4 reveal a quantitative relationship between caffeine affinities and the measured solubility values. It is worth mentioning that values of Δ*G_r_* used for plots preparation correspond to concentration-dependent equilibrium constants (*K_x_*) rather than the activity ones (*K_a_*), which were included in Table 1. By separating mole fraction contributions to equilibrium constants from contributions coming from the activity coefficient it is also possible to inspect how the concentration of the solvent affects the affinity Δ*G_r_^(x)^*, as seen in Figure 5. It seems that there is a correlation between experimental solubility and solute–solvent affinities with acceptable linearity (R^2^ = 0.78) of this relationship. Hence, Figure 4 further documents the importance of direct contacts of caffeine, although the existence of linear relationships is not so obvious taking into account that only pairs are included in the solubility modeling. Caffeine dimerization, although much less favorable under all studied conditions, also exhibits a linear relationship with respect to experimental solubility. However, total intermolecular interaction including all possible contacts is a much less reliable prognostic for solubility predictions. Probably including many potential multi-molecular complexes in the model would be beneficial. Unfortunately, this is prevented not only by the number of potential structures to be considered but also by the expense of computations, which include not only zero-point energy correction but also encompass electron correlation and dispersion corrections.

### 3.5. Screening of Caffeine Solubility

**Figure 6:**
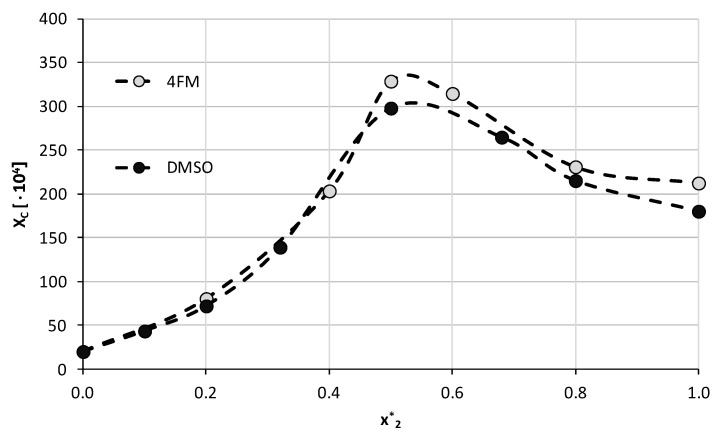
Experimental solubility of caffeine, expressed as its mole fractions, in binary solvents comprising water and 4-formylmorpholine in varying compositions. On the abscissa, x^*^_2_ represents the mole fractions of organic solvent in solute-free binary solutions. Temperature was set to 25 °C and solubility values obtained in DMSO were shown for comparison.

The observation of a semi-quantitative linear correlation of caffeine solubility in systems comprising apolar solvents with computed solute–solvent affinities offers the possibility of screening for new solvents belonging to the same class. The set of organic solvents used in this paper consists of commonly used ones. Although their properties are well recognized, some of their features are often ignored, such as, for example, environmental impact. The most spectacular example is DMSO. It is generally considered a green, safe and non-toxic solvent. However, according to classification by the U.S. Environmental Protection Agency (EPA) [96], the overall environmental index (EI) is as high as 11.68. This index is defined as a sum of many contributions including scores characterizing Human Toxicity Potential by Ingestion, by Inhalation, Terrestrial Toxicity Potential, Aquatic Toxicity Potential, Global Warming Potential, Ozone Depletion Potential, Photochemical Oxidation Potential (PCOP) and Acid Rain Potential [97]. In the case of DMSO, the source of such a high value of EI originates from the very high PCOP contribution. This parameter quantifies the stability of a given compound by comparing the rate of a chemical reaction with a hydroxyl radical to the rate of an analogical reaction of ethylene. In the case of DMSO, this parameter has a dominant influence on the overall value of EI. After excluding this score from summation, the obtained environmental index drops down to EI(PCOP = 0) = 0.26 suggesting the greenness of DMSO. However, with DMSO and DMF, there are several associated warnings such as potential source of skin irritation, serious eye irritation, possible respiratory irritation and also specific toxicity for many organs. Acetone, acetonitrile and 1,4-dioxane, apart from these warnings, are also flammable and harmful if swallowed. Hence, it is of crucial importance to also include the potential environmental impact in the solubility analysis and such information regarding these common solvents is provided in Table 2. Nowadays [80], screening for the most efficient and, at the same time, green solvents for sulfamethizol turned our attention toward morpholine analogs, among which many can be considered as APAS. A short list of such potential solvents is also provided in Table 2. The list was restricted only to such solvents which are aprotic and greener when compared to common solvents. It is worth noting that 4-formylmorpholine (4FM) is characterized by the smallest value of EI and seems to be the first choice alternative to studied solvents. Taking advantage of the linear relationship between solubility and solute–solvent affinities it is possible to predict solubility in new solvents. Even though only a modest linear relationship is observed, the qualitative agreement allows for narrowing the list of solvents potentially interesting for experimental solubility determination. The results of affinity computations for selected morpholines are collected in Table 3. It is directly visible that the structural motif of solute–solvent contacts formation resembles the ones previously observed for other APAS. Both the geometrical parameters of hydrogen bonding and the energetics of pair formation with caffeine suggest that all morpholines can act as effective solvents. Interestingly, the affinities of methyl and ethyl analogues are slightly higher compared to 4FM and morpholine. As it was evidenced in Table 2, 4-formylmorpholine is the most interesting solvent due to the lowest value of EI. Hence, 4FM was selected for testing the presented above hypothesis that the affinity of solute can be a valuable and direct guidance for selection of alternative solvents. Thus, caffeine solubility in 4FM was measured for quantification of the solubilizing potential of this APAS. The results obtained at 25 °C are collected in Figure 6, with corresponding details available in the Supplementary Materials, Table S8. For comparison purposes, the solubility of caffeine in DMSO is also provided in the figure.

The general trend of caffeine solubility in solutions comprising the selected solvent and water in varying mole compositions is analogical to the ones obtained earlier. The highest solubility, i.e., x_C_ = (325.52 ± 4.58)∙10^−4^ was found for the composition with unimolar proportions of both binary solvent constituents. Using pure 4FM as the solvent resulted in the solubility of caffeine equal x_C_ = (212.80 ± 1.53)∙10^−4^. The obtained results indicate that this organic solvent is more effective than DMSO with caffeine solubility increased by 10.3% in the optimal composition and by 17.9% in the pure solvent. This observation confirms the usefulness of the described screening procedure in finding suitable candidates for caffeine dissolution in terms of both efficiency and environmental friendliness.

## 4. Conclusions

The solubility of caffeine was studied in aqueous binary mixtures of some aprotic proton acceptor solvents (APAS). The performed experiments revealed that adding a specific amount of water to the studied organic solvents resulted in achieving a cosolvation effect and ultimately in an increase of caffeine solubility compared to neat solvents. In all cases, a unimolar amount of water and organic solvent was responsible for the largest obtained solubility of caffeine. The highest solubilization efficiency was observed for the DMSO–water system and in the optimal composition at 40 °C the caffeine solubility was found to be x_C_ = (425.89 ± 0.92)∙10^−4^, which amounts to about 65% increase compared to neat DMSO. The order of decreasing solubilizing potential of binary systems containing water and organic solvents is as follows: DMSO > DMF > 1,4-dioxane > acetonitrile > acetone. A general trend shows that smaller caffeine solubilities in neat solvents are associated with a higher solubility gain in the optimal compositions.

In order to interpret the observed solubility trends, the affinities of caffeine toward hetero-molecular pairs formation were studied using advanced quantum chemistry computations. It was found that caffeine can act as a donor in pairs formation with all considered APAS. This is due to the hydrogen atom bound to the carbon atom within the five-membered ring (C_8_), which thanks to its electropositive character can be involved in interactions with negative centers located on oxygen atoms of solvent molecules, which can be considered as weak hydrogen bonds. There was found an interesting correspondence between the stabilization of the formed pairs and the observed experimental solubility, namely the larger stabilization was associated with higher solubility of caffeine. Furthermore, the knowledge about the electropositive region in a solute molecule can be used to anticipate interactions with apolar solvents containing negative centers. The computed values of Gibbs free energies of self-association and intermolecular pairs formation of solute–solute, solute–solvent and solvent–solvent interactions were further studied for exploring the possibility of using them as potential solubility prognostics. A semi-quantitative relationship between caffeine affinities and the measured solubility values was found, with the highest linearity (R^2^ = 0.78) observed in the case of caffeine–solvent interactions.

The observed fact that caffeine solubility in systems comprising apolar solvents is correlated with computed solute–solvent affinities enables screening for new solvents of the same class. The environmental impact of many solvents is a factor limiting their applications and therefore there is a need for alternative ones. The environmental index (EI) offered by the U.S. Environmental Protection Agency (EPA) is a measure of environmental impact used for suggesting solvents for caffeine dissolution alternative to DMSO. Based on previous experience, morpholine analogs, among which many can be considered as aprotic solvents, were suggested as an interesting alternative. A list of potential solvents was created and, based on their EI indices, the 4-formylmorpholine was selected for solubility experiments. It turned out that this solvent was even more effective in caffeine dissolution than DMSO both in the neat form, as well as in a binary mixture in unimolar proportions with water. The obtained result shows the usefulness of such types of screening in the search for environmentally-friendly replacements of traditional solvents.

## Figures and Tables

**Table 1:** Characteristics of the most stable bimolecular hetero-molecular complexes formed by caffeine in analyzed solutions. Primes in the geometry parameters denote atoms of solvent molecules. The values of Gibbs free energy of reaction represent the concentration-independent quantity derived using activities at 25 °C.

**caffeine–DMF**	**caffeine–DMSO**		
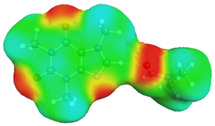 d_H__⋯__O’_ = 2.149 Å, α_C-H__⋯__O’_ = 176.3° ΔG_r_(AB) = −5.7 kcal/mol	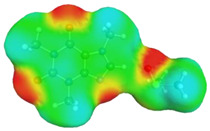 d_H__⋯O’_= 2.105 Å, α_C-H__⋯O’_= 179.1° ΔG_r_(AB) = −6.5 kcal/mol	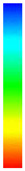	+0.01e^2^/Å^2^ −0.01e^2^/Å^2^
**caffeine–1,4-dioxane**	**caffeine–acetone**		
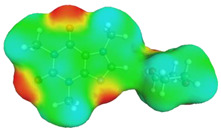 d_H__⋯O’_ = 2.192 Å, α_C-H__⋯O’_ = 176.5° ΔG_r_(AB) = −5.0 kcal/mol	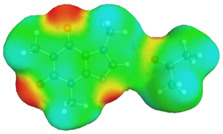 d_H__⋯O’_ = 2.283 Å, α_C-H__⋯O’_ = 175.5° ΔG_r_(AB) = −4.2 kcal/mol		
**caffeine–acetonitrile**	**caffeine–water**		
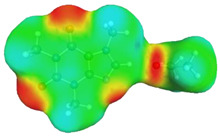 d_H__⋯O’_ = 2.388 Å, α_C-H__⋯O’_ = 175.5° ΔG_r_(AB) = −3.5 kcal/mol	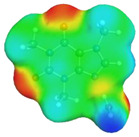 d_N__⋯H’_ = 1.852 Å, α_C-H__⋯O’_ = 177.3° ΔG_r_(AB) = −6.4 kcal/mol		

**Table 2:** Collection of environmental indices of common solvents used in this study and some morpholine derivatives.

Solvent	CAS	EI	EI_(PCOP=0)_	T_B_ [K]
DMSO	[67-68-5]	11.68	0.26	462
DMF	[68-12-2]	2.16	2.16	426
1,4-dioxane	[123-91-1]	5.26	0.97	375
acetone	[67-64-1]	1.27	0.66	329
acetonitrile	[75-05-8]	1.88	1.88	355
morpholine	[110-91-8]	2.95	2.95	401
4-formylmorpholine	[4394-85-8]	0.51	0.51	512
4-methylmorpholine	[109-02-4]	1.93	1.93	389
4-ethylmorpholine	[100-74-3]	2.12	2.12	412

**Table 3:** Characteristics of the most stable bimolecular hetero-molecular complexes formed by caffeine with morpholine and its three N-substituted analogs. Primes in the geometry parameters denote atoms of solvent molecules. The values of Gibbs free energy of reaction represent the concentration-independent quantity derived using activities at 25 °C.

**caffeine–morpholine** ΔGr(AB) = −5.2 kcal/mol			
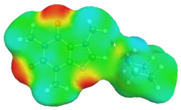 d_H__⋯N’_ = 2.227 Å, α_C-H__⋯N’_ = 179.2°	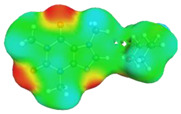 d_H__⋯O’_ = 2.165 Å, α_C-H__⋯O’_ = 178.7°	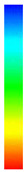	+0.01e^2^/Å^2^ −0.01e^2^/Å^2^
**caffeine–4-formylmorpholine** ΔG_r_(AB) = −5.8 kcal/mol			
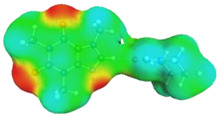 d_H__⋯O’_ = 2.159 Å, α_C-H__⋯O’_ = 177.9°	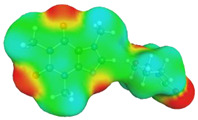 d_H__⋯O’_ = 2.226 Å, α_C-H__⋯O’_ = 173.9°		
**caffeine–4-methylmorpholine** ΔG_r_(AB) = −6.7 kcal/mol			
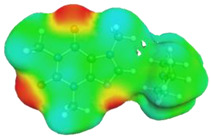 d_H__⋯N’_ = 2.313 Å, α_C-H__⋯N’_ = 172.8°	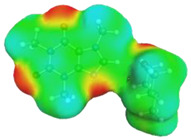 d_H__⋯O’_ = 2.221 Å, α_C-H__⋯O’_ = 178.5°		
**caffeine–4-ethylmorpholine** ΔG_r_(AB) = −6.8 kcal/mol			
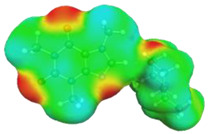 d_H__⋯O’_ = 2.204 Å, α_C-H__⋯O’_ = 175.5°	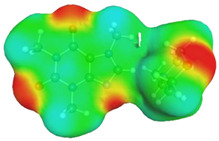 d_H__⋯N’_ = 2.358 Å, α_C-H__⋯N’_ = 171.3°		

## Data Availability

Not applicable.

## Supplementary Materials

The following supporting information can be downloaded at: https://www.mdpi.com/article/10.3390/ma15072472/s1, Figure S1. The FTIR spectra of caffeine precipitates obtained after solubility measurements in DMSO, water and their mixtures. Values in the legend indicate the mole fractions of DMSO in solute-free binary solvents. For comparison, the spectrum of pure caffeine is shown. Figure S2. The DSC curves of caffeine precipitates obtained after solubility measurements in DMSO, water and their mixtures. Values in the figure indicate the mole fractions of DMSO in solute-free binary solvents. For guiding the eye vertical lines are drawn corresponding to the hydrate water loss (dashed line) and the transition from form II to form I (dotted line). For comparison, the DSC curve of pure caffeine is also provided. Table S1a. Concentrations of caffeine solutions and the corresponding absorbance values, together with mean absorbance and standard deviation (SD) values, used during preparation of the calibration curve. Table S1b. Parameters of the obtained calibration curve for caffeine solubility determination. Table S2. Comparison of caffeine solubility values expressed as mole fractions (∙10^4^) obtained in this study and the results taken from literature. Standard deviation values (∙10^4^) are given in parentheses. Relative differences between datasets are also provided. Table S3. Mole fractions (∙10^4^) and standard deviation (SD) values (∙10^4^) of caffeine in binary solvents comprising water and DMSO in different proportions. Table S4. Mole fractions (∙10^4^) and standard deviation (SD) values (∙10^4^) of caffeine in binary solvents comprising water and DMF in different proportions. Table S5. Mole fractions (∙10^4^) and standard deviation (SD) values (∙10^4^) of caffeine in binary solvents comprising water and dioxane in different proportions. Table S6. Mole fractions (∙10^4^) and standard deviation (SD) values (∙10^4^) of caffeine in binary solvents comprising water and acetone in different proportions. Table S7. Mole fractions (∙10^4^) and standard deviation (SD) values (∙10^4^) of caffeine in binary solvents comprising water and acetonitrile in different proportions. Table S8. Mole fractions (∙10^4^) and standard deviation (SD) values (∙10^4^) of caffeine in binary solvents comprising water and 4-formylmorpholine in different proportions at 25 °C.
