# Supplementary material for: Application of the Solute–Solvent Intermolecular Interactions as Indicator of Caffeine Solubility in Aqueous Binary Aprotic and Proton Acceptor Solvents: Measurements and Quantum Chemistry Computations
Source: Materials (Basel). 2022 Mar 27;15(7):2472. doi: 10.3390/ma15072472 (PMC8999965; doi:10.3390/ma15072472)
Supplement: Supplementary file 1 [file materials-15-02472-s001.zip › materials-1633896-supplementary.pdf]

Application of the Solute-Solvent Intermolecular Interactions as Indicator of Caffeine Solubility in Aqueous Binary Aprotic and Proton Acceptor Solvents: Measurements and Quantum Chemistry Computations

Tomasz Jeliński [1],*, Maciej Kubsik [1] and Piotr Cysewski [1],*

1 Department of Physical Chemistry, Pharmacy Faculty, Collegium Medicum of Bydgoszcz, Nicolaus Copernicus University in Toruń, Kurpińskiego 5, 85-950 Bydgoszcz, Poland

* Correspondence: tomasz.jelinski@cm.umk.pl (T.J.); piotr.cysewski@cm.unk.pl (P.C.)

Supplementary Materials

Figure S1. The FTIR spectra of caffeine precipitates obtained after solubility measurements in DMSO, water and their mixtures. Values in the legend indicate the mole fractions of DMSO in solute-free binary solvents. For comparison, the spectrum of pure caffeine is shown.

Figure S2. The DSC curves of caffeine precipitates obtained after solubility measurements in DMSO, water and their mixtures. Values in the figure indicate the mole fractions of DMSO in solute-free binary solvents. For guiding the eye vertical lines are drawn corresponding to the hydrate water loss (dashed line) and the transition from form II to form I (dotted line). For comparison, the DSC curve of pure caffeine is also provided.

Table S1a. Concentrations of caffeine solutions and the corresponding absorbance values, together with mean absorbance and standard deviation (SD) values, used during preparation of the calibration curve.

Table S1b. Parameters of the obtained calibration curve for caffeine solubility determination.

Table S2. Comparison of caffeine solubility values expressed as mole fractions ($\cdot 10^4$) obtained in this study and the results taken from literature. Standard deviation values ($\cdot 10^4$) are given in parentheses. Relative differences between datasets are also provided.

Table S3. Mole fractions ($\cdot 10^4$) and standard deviation (SD) values ($\cdot 10^4$) of caffeine in binary solvents comprising water and DMSO in different proportions.

Table S4. Mole fractions ($\cdot 10^4$) and standard deviation (SD) values ($\cdot 10^4$) of caffeine in binary solvents comprising water and DMF in different proportions.

Table S5. Mole fractions ($\cdot 10^4$) and standard deviation (SD) values ($\cdot 10^4$) of caffeine in binary solvents comprising water and dioxane in different proportions.

Table S6. Mole fractions ($\cdot 10^4$) and standard deviation (SD) values ($\cdot 10^4$) of caffeine in binary solvents comprising water and acetone in different proportions.

Table S7. Mole fractions ($\cdot 10^4$) and standard deviation (SD) values ($\cdot 10^4$) of caffeine in binary solvents comprising water and acetonitrile in different proportions.

Table S8. Mole fractions ($\cdot 10^4$) and standard deviation (SD) values ($\cdot 10^4$) of caffeine in binary solvents comprising water and 4-formylmorpholine in different proportions at 25°C.

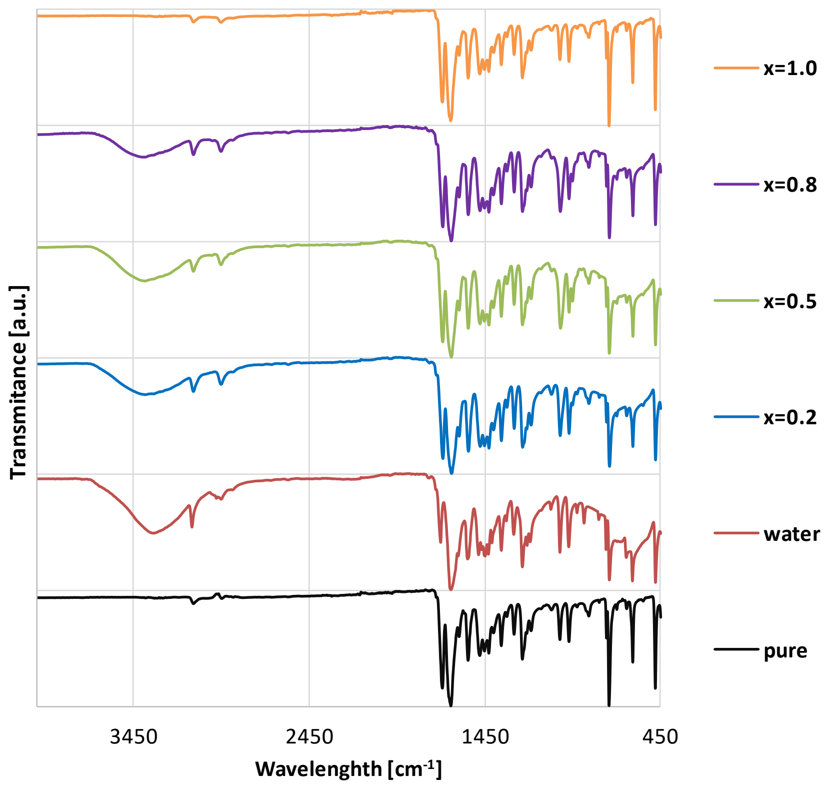

Figure S1. The FTIR spectra of caffeine precipitates obtained after solubility measurements in DMSO, water and their mixtures. Values in the legend indicate the mole fractions of DMSO in solute-free binary solvents. For comparison, the spectrum of pure caffeine is shown.

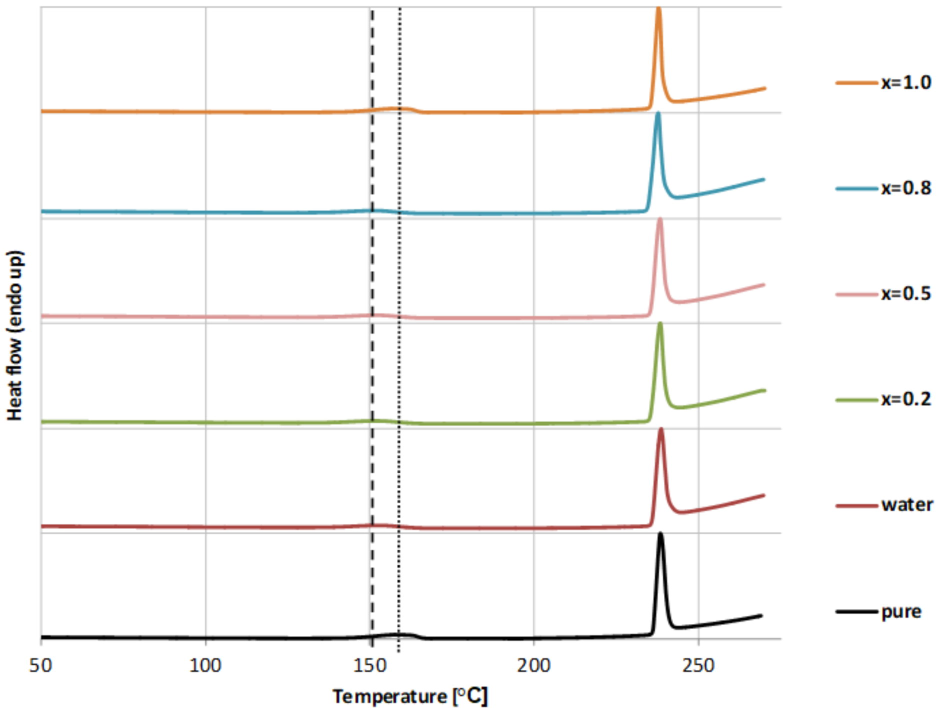

Figure S2. The DSC curves of caffeine precipitates obtained after solubility measurements in DMSO, water and their mixtures. Values in the figure indicate the mole fractions of DMSO in solute-free binary solvents. For

guiding the eye vertical lines are drawn corresponding to the hydrate water loss (dashed line) and the transition from form II to form I (dotted line). For comparison, the DSC curve of pure caffeine is also provided.

Table S1a. Concentrations of caffeine solutions and the corresponding absorbance values, together with standard deviation (SD) values, used during preparation of the calibration curve.

c [mg/ml]	A_{mean}	SD_A
0.03230	2.428	0.030
0.02584	1.942	0.038
0.02153	1.634	0.017
0.01846	1.398	0.020
0.01615	1.219	0.002
0.01436	1.074	0.018
0.01292	0.972	0.017
0.01175	0.896	0.023
0.01077	0.814	0.015
0.00994	0.733	0.015
0.00923	0.695	0.030
0.00861	0.648	0.023

Table S1b. Parameters of the obtained calibration curve.

parameter	value
a	75.338
b	$4.441 \cdot 10^{-16}$
R^2	0.999
LOD	$3{,}646 \cdot 10^{-4}$ mg/ml
LOQ	$1{,}094 \cdot 10^{-3}$ mg/ml

Table S2. Comparison of caffeine solubility values expressed as mole fractions ($\cdot 10^4$) obtained in this study and the results taken from literature. Standard deviation values ($\cdot 10^4$) are given in parentheses. Relative differences between datasets are also provided.

dataset	15°C	25°C	30°C	35°C	40°C	45°C	55°C
				**water**			
this study	-	20.2(±0.2)	25.6(±0.2)	31.6(±0.1)	38.3(±0.3)	-	-
Zhong 2017 [1]	12.3(±0.1)	16.1(±0.4)	-	22.7(±0.4)	-	29.6(±0.8)	39.8(±0.8)
		25.4%	-	*39.4%*			
Shalmashi 2010 [2]	-	21.0(±0.1)	26.2(±0.2)	30.8(±0.1)	43.7(±0.6)	79.2(±0.1)	-
		-3.8%	*-2.5%*	*2.9%*	*-7.6%*		
Dabir 2018 [3]	-	19.3(±0.6)	25.4(±0.8)	31.4(±0.9)	41.4(±1.2)	-	-
		4.6%	*0.6%*	*0.8%*	*-7.6%*		
				**methanol**			
this study	-	20.8(±0.1)	26.3(±0.3)	33.0(±0.5)	41.6(±0.2)	-	-
Zhong 2017 [1]	13.4(±0.1)	18.9(±0.2)	-	28.0(±0.3)	-	42.6(±0.1)	66.3(±1.1)
		9.8%		*17.9%*			
Shalmashi 2010 [2]	-	20.0(±0.1)	26.4(±0.1)	34.1(±0.1)	41.9(±0.1)	-	-

		3.5%	*-0.1%*	*-3.2%*	*-0.7%*		
				**ethanol**			
this study		16.0(±0.1)	19.5(±0.3)	22.7(±0.1)	26.6(±0.2)	-	-
Zhong 2017 [1]	7.8(±0.2)	13.2(±0.1)	-	20.4(±0.1)	-	32.2(±0.2)	46.9(±0.4)
		21.5%		*11.2%*			
Shalmashi 2010 [2]	-	17.1(±0.1)	20.5(±0.1)	21.9(±0.1)	26.5(±0.1)	-	-
		-6.3%	*-4.6%*	*3.7%*	*0.8%*		
				**ethyl acetate**			
this study	-	38.3(±0.2)	43.3(±0.7)	49.7(±0.4)	57.7(±0.7)	-	-
Zhong 2017 [1]	27.0(±0.5)	35.4(±0.1)	-	48.8(±0.6)	-	68.9(±2.1)	92.4(±3.3)
		4.8%		*1.1%*			
Shalmashi 2010 [2]	-	40.5(±0.1)	44.6(±0.1)	48.4(±0.2)	55.2(±0.3)	-	-
		-5.3%	*-3.0%*	*2.7%*	*4.4%*		
				**acetone**			
this study		42.0(±0.5)	54.1(±0.7)	65.6(±0.4)	78.0(±0.6)	-	-
Zhong 2017 [1]	27.9(±0.3)	36.3(±0.9)	-	52.8(±0.2)	-	72.8(±1.1)	95.3(±1.3)
		15.8%		*24.2%*			
Shalmashi 2010 [2]	-	45.4(±0.1)	57.6(±0.1)	69.8(±0.1)	84.7(±0.2)	-	-
		-7.3%	*-6.1%*	*-6.0%*	*-7.9%*		
				**1-propanol**			
this study	-	18.6(±0.1)	22.9(±0.6)	28.5(±0.2)	35.8(±0.4)	-	-
Zhong 2017 [1]	11.7(±0.2)	17.7(±0.2)	-	28.2(±0.1)	-	45.3(±0.7)	71.6(±1.3)
		4.8%		*1.1%*			

[1] Zhong, J.; Tang, N.; Asadzadeh, B.; Yan, W. Measurement and Correlation of Solubility of Theobromine, Theophylline, and caffeine in Water and Organic Solvents at Various Temperatures. *J. Chem. Eng. Data* **2017**, *62*, 2570–2577.
[2] Shalmashi, A.; Golmohammad, F. Solubility of caffeine in water, ethyl acetate, ethanol, carbon tetrachloride, methanol, chloroform, dichloromethane, and acetone between 298 and 323 K. *Lat. Am. Appl. Res.* **2010**, *40*, 283–285.
[3] Dabir, T.O.; Gaikar, V.G.; Jayaraman, S.; Mukherjee, S. Thermodynamic modeling studies of aqueous solubility of caffeine, gallic acid and their cocrystal in the temperature range of 303 K–363 K. *Fluid Phase Equilib.* **2018**, *456*, 65–76.

Table S3. Mole fractions (·10^4) and standard deviation (SD) values (·10^4) of caffeine in binary solvents comprising water and DMSO in different proportions.

x_{DMSO}	**25°C**		**30°C**		**35°C**		**40°C**	
	**x_C(·10^4)**	**SD(·10^4)**	**x_C(·10^4)**	**SD(·10^4)**	**x_C(·10^4)**	**SD(·10^4)**	**x_C(·10^4)**	**SD(·10^4)**
0.00	20.195	0.222	25.567	0.178	31.658	0.116	38.264	0.290
0.03	30.523	0.242	35.917	0.139	42.444	0.132	49.432	0.307
0.07	36.368	0.131	42.832	0.227	49.340	0.181	56.446	0.253
0.10	43.651	0.286	48.839	0.225	55.668	0.207	62.120	0.349
0.13	49.986	0.516	56.306	0.277	63.967	0.405	72.267	0.163
0.17	59.656	0.316	68.240	0.247	78.298	0.411	87.267	0.481
0.20	72.260	0.177	82.388	0.501	96.057	0.613	108.665	0.511
0.32	139.337	0.500	154.334	0.330	179.281	0.753	200.653	0.986
0.50	298.190	1.104	332.805	0.867	384.165	1.737	425.895	0.917

0.68	275.454	0.640	312.867	0.389	357.740	2.183	408.021	1.723
0.80	215.364	1.349	240.656	0.802	272.044	1.640	309.820	1.493
1.00	180.388	2.127	200.314	36.492	228.354	1.782	257.715	1.211

Table S4. Mole fractions (·10⁴) and standard deviation (SD) values (·10⁴) of caffeine in binary solvents comprising water and DMF in different proportions.

x_{DMF}	25°C		30°C		35°C		40°C	
	x_C(·10⁴)	SD(·10⁴)	x_C(·10⁴)	SD(·10⁴)	x_C(·10⁴)	SD(·10⁴)	x_C(·10⁴)	SD(·10⁴)
0.00	20.195	0.222	25.567	0.178	31.658	0.116	38.273	0.290
0.03	27.335	0.054	32.944	0.219	39.927	0.236	46.852	0.225
0.07	33.005	0.246	38.818	0.388	46.141	0.206	56.057	0.262
0.10	36.310	0.159	44.209	0.411	51.093	0.177	61.913	0.302
0.13	42.732	0.158	50.319	0.739	58.502	0.203	69.645	0.555
0.17	53.095	0.244	62.416	0.425	73.844	0.418	88.087	0.630
0.20	65.661	0.431	77.743	0.569	90.652	0.759	109.488	0.525
0.32	114.384	0.427	132.199	1.167	153.683	0.852	176.906	1.804
0.50	251.926	1.025	288.839	2.603	334.765	1.777	383.958	2.166
0.68	234.439	1.264	269.503	1.545	310.970	2.020	364.995	3.099
0.80	176.316	1.062	202.810	1.245	233.622	1.453	275.303	2.192
1.00	129.419	0.789	147.268	0.958	172.285	0.685	202.167	0.814

Table S5. Mole fractions (·10⁴) and standard deviation (SD) values (·10⁴) of caffeine in binary solvents comprising water and 1,4-dioxane in different proportions.

$x_{dioxane}$	25°C		30°C		35°C		40°C	
	x_C(·10⁴)	SD(·10⁴)	x_C(·10⁴)	SD(·10⁴)	x_C(·10⁴)	SD(·10⁴)	x_C(·10⁴)	SD(·10⁴)
0.00	20.195	0.222	25.567	0.178	31.658	0.116	38.273	0.290
0.03	24.560	0.101	29.686	0.135	36.596	0.184	44.030	0.183
0.07	28.013	0.134	33.845	0.074	39.276	0.376	46.641	0.478
0.10	32.165	0.358	38.792	0.303	45.164	0.344	54.572	0.475
0.13	36.757	0.173	43.520	0.310	50.487	0.390	59.107	0.515
0.17	43.518	0.392	50.473	0.292	59.369	0.289	70.427	0.466
0.20	50.059	0.378	58.617	0.188	68.720	0.299	80.817	0.745
0.32	96.607	0.322	111.693	0.478	128.386	1.463	148.620	1.379
0.50	177.437	1.246	201.037	1.226	233.550	2.096	269.769	2.593
0.68	145.232	0.900	168.777	1.544	192.378	1.876	226.973	2.015
0.80	84.880	0.875	100.175	0.467	118.932	0.606	140.045	0.733
1.00	50.688	0.229	60.176	0.116	71.059	0.444	84.274	0.601

Table S6. Mole fractions (·10⁴) and standard deviation (SD) values (·10⁴) of caffeine in binary solvents comprising water and acetone in different proportions.

$x_{acetone}$	25°C		30°C		35°C		40°C	
	x_C(·10⁴)	SD(·10⁴)	x_C(·10⁴)	SD(·10⁴)	x_C(·10⁴)	SD(·10⁴)	x_C(·10⁴)	SD(·10⁴)
0.00	20.195	0.222	25.567	0.178	31.658	0.116	38.273	0.290
0.03	22.502	0.261	26.150	0.127	32.593	0.122	40.025	0.347
0.07	26.531	0.160	30.698	0.218	36.546	0.280	44.612	0.527
0.10	31.372	0.277	35.550	0.231	43.147	0.128	51.732	0.378
0.13	35.921	0.264	44.641	0.354	52.437	0.287	62.289	0.666

0.17	45.139	0.279	54.260	0.163	63.640	0.416	76.476	0.763
0.20	54.190	0.292	62.923	0.358	74.969	0.586	92.480	0.514
0.32	95.177	0.457	112.863	0.753	129.552	0.683	152.248	1.023
0.50	116.329	0.605	133.064	0.563	157.787	0.625	186.505	0.951
0.68	84.181	0.622	96.898	1.290	114.404	1.056	135.685	1.212
0.80	57.178	0.425	66.831	0.544	77.880	0.357	90.773	0.458
1.00	42.032	0.515	54.098	0.779	65.560	0.336	77.996	0.556

Table S7. Mole fractions ($\cdot 10^4$) and standard deviation (SD) values ($\cdot 10^4$) of caffeine in binary solvents comprising water and acetonitrile in different proportions.

$x_{acetonitrile}$	25°C		30°C		35°C		40°C	
	$x_C(\cdot 10^4)$	SD($\cdot 10^4$)	$x_C(\cdot 10^4)$	SD($\cdot 10^4$)	$x_C(\cdot 10^4)$	SD($\cdot 10^4$)	$x_C(\cdot 10^4)$	SD($\cdot 10^4$)
0.00	20.195	0.222	25.567	0.178	31.650	0.116	38.273	0.290
0.03	21.445	0.227	26.399	0.302	32.573	0.603	40.176	0.401
0.07	24.487	0.302	29.309	0.210	35.781	0.499	43.960	0.372
0.10	28.007	0.241	34.060	0.227	40.500	0.575	50.355	0.510
0.13	34.243	0.142	40.656	0.210	47.265	0.579	56.800	0.275
0.17	42.544	0.275	49.987	0.456	59.391	0.488	69.938	0.428
0.20	54.991	0.309	66.214	0.472	77.142	0.665	91.071	1.114
0.32	98.713	0.856	114.162	0.788	137.215	0.891	160.668	2.288
0.50	128.131	1.086	145.064	0.650	172.590	0.206	202.404	1.880
0.68	87.198	0.728	100.024	0.335	119.029	2.036	141.704	1.958
0.80	54.808	0.710	63.466	0.753	74.075	0.887	88.250	0.867
1.00	30.488	0.099	35.317	0.554	40.920	0.373	47.058	0.498

Table S8. Mole fractions ($\cdot 10^4$) and standard deviation (SD) values ($\cdot 10^4$) of caffeine in binary solvents comprising water and 4-formylmorpholine in different proportions at 25°C.

x_{4FM}	$x_C(\cdot 10^4)$	SD($\cdot 10^4$)
0.00	20.195	0.222
0.20	80.583	0.916
0.40	203.614	1.749
0.50	328.969	2.797
0.60	314.865	4.586
0.80	231.141	4.443
1.00	212.612	1.531
